# The menstrual phase does not impact chemosensitivity during exercise

**DOI:** 10.14814/phy2.70169

**Published:** 2024-12-23

**Authors:** Leah M. Mann, Madeline D. Wright, Benjamin P. Thompson, Jou‐Chung Chang, Jason S. Chan, Glen E. Foster, Paolo B. Dominelli

**Affiliations:** ^1^ Department of Kinesiology and Health Sciences University of Waterloo Waterloo Ontario Canada; ^2^ School of Health and Exercise Sciences The University of British Columbia Kelowna British Columbia Canada; ^3^ Faculty of Kinesiology University of Calgary Kelowna Calgary, Alberta Canada

**Keywords:** chemosensitivity, end‐tidal forcing, exercise, menstrual cycle

## Abstract

At rest, the menstrual cycle phase impacts ventilation and chemosensitivity. However, during exercise there is inconclusive evidence that the menstrual cycle phase affects ventilation or chemosensitivity. We sought to examine the influence of menstrual phase and hormonal birth control (BC) on chemosensitivity. We tested 12 males and 20 females (10 BC; 10 normally menstruating, NBC) on three occasions. Day 1 was a maximal exercise test and days 2 (follicular phase) and 3 (luteal phase) consisted of three bouts of chemosensitivity testing during cycle exercise at 30% of peak work rate. Females‐BC and males completed day 3 approximately 2 weeks after day 2, with females‐BC tested during the active phase of their birth control. There were no differences between the two experimental days for any groups for any (hypercapnia, hypoxia, and hyperoxia) chemosensitivity tests, *p* > 0.05. Females‐BC had a significantly lower average response to transient hypercapnia than both females‐NBC and males (38% and 42% lower, respectively, *p* < 0.05). Females‐NBC had a significantly smaller change in ventilation to hyperoxia compared to males, −11.7 ± 5.9 versus −17.9 ± 5.4%, respectively (*p* < 0.05). We conclude that the day‐to‐day variability in chemosensitivity is not different between males, females‐BC and NBC.

## 1 INTRODUCTION

Over the course of the menstrual cycle, significant hormonal changes cause fluctuations in physiological responses, such as increased resting ventilation during the luteal phase (Dombovy et al., 1987; MacNutt et al., 2012; Schoene et al., 1981). Ventilatory control comes, in part, from central and peripheral chemoreceptors that respond to changes in arterial partial pressure of carbon dioxide (PaCO_2_), oxygen (PaO_2_), as well as other stimuli. Hypercapnia and hypoxia both stimulate an increase in ventilation, allowing for individual measures of the peripheral chemoreceptor activity to these stimuli, while transient hyperoxia decreases ventilation and can test the tonic input of the peripheral chemoreceptors (Blain et al., 2009).

The potential link between the menstrual cycle and the chemoresponse has been examined previously, and progesterone is implicated to act peripherally (on the carotid chemosensor) to increase the hypoxic and hypercapnic sensitivity (Hannhart et al., 1990; Slatkovska et al., 2006; Tatsumi et al., 1997). For example, there appears to be an effect of sex as chemosensitivity was lower in females relative to males at rest (Jensen et al., 2005; MacNutt et al., 2012), regardless of menstrual phase. However, the hypercapnic ventilatory response at rest has also been shown to be increased during the luteal phase compared to the follicular phase in normally menstruating females, whereas those experiencing amenorrhea had no change in the hypercapnic ventilatory response between days (Schoene et al., 1981). There is also an increase in the ventilatory response to CO_2_ within individuals using hormonal contraceptives compared to when they are not taking them (Smith & Mines, 1982). Exogenous progesterone can also increase ventilation in males, at rest and during exercise, similar to the effects of the luteal phase in menstruating females (Bonekat et al., 1987), with similar results being shown in male rats, where progesterone caused an increased ventilation response to hypoxia (Tatsumi et al., 1993). While studies at rest highlight variability in chemoreception in normally menstruating females during different phases, during exercise the menstrual cycle phase has been shown to have little to no effect on exercise performance in the absence of hypercapnia (Carmichael et al., 2021; Smekal et al., 2007; Taylor et al., 2024). While the hypercapnic ventilatory response in normally menstruating females during exercise was not different regardless of menstrual cycle phase (Itoh et al., 2007), the potential effect on the hypoxic ventilatory response is inconclusive (Dombovy et al., 1987; MacNutt et al., 2012; Schoene et al., 1981).

We recently demonstrated the peripheral hypercapnic response is increased during exercise and greater in males compared to females; however, the response does not continue to increase with exercise intensity regardless of sex (Mann et al., 2022; Wright et al., 2023). The within‐cycle variations were unknown because menstrual cycle phase was not reported. A potential hypothesis is that the influence of feedforward/feedback mechanisms during exercise, like the metaboreflex and mechanoreflex from exercising muscles, represent a greater control stimulus and may minimize any changes in ventilation or chemosensitivity arising from the menstrual cycle; however, at rest some research suggests that the metaboreflex and mechanoreflex are hyperadditive with the chemoreflex (de Oliveira et al., 2023; Edgell & Stickland, 2014).

A caveat to previous chemoreceptor testing during exercise is the interplay between carbon dioxide and oxygen that causes opposing changes in ventilation. Specifically, when testing the hypoxic ventilatory response, ventilation increases and there is a decrease in arterial CO_2_ levels, which restrains the ventilatory response (Steinback & Poulin, 2007). As such, to precisely determine the potential effect of the menstrual cycle on the chemoresponse during exercise, testing should be performed under isocapnic or iso‐oxic conditions.

Thus, we sought to investigate the variability of the peripheral and central chemoreceptors response using transient and iso‐oxic steady state hypercapnia, transient hyperoxia, and isocapnic hypoxia testing during submaximal exercise in females who were normally menstruating or using a hormonal birth control drug, and males. By testing normally menstruating females at both the early follicular and early luteal phases, we can better understand how menstrual cycle phase may impact the variability of the chemoresponses in this population compared to males and females on hormonal birth control. Our study is not designed to test the role of absolute hormone concentrations on the exercise chemoresponse; instead, we have chosen distinct time points where an individual's hormone profile is likely to be the same (e.g., using birth control) or different (e.g., menstrual phases) to determine if this causes a greater degree of variability. Understanding how the variability of the chemoresponses change during exercise in males, females using hormonal birth control (females‐BC) and females normally menstruating and not using hormonal birth control (females‐NBC) will inform future research by increasing female inclusivity. Our primary hypothesis was that there would be no difference in the exercise chemoresponse between the luteal phase and the follicular phase for females‐NBC, with a secondary hypothesis that females‐BC and males would also not have a different chemosensitivity between days.

## 2 METHODS

### 2.1 Ethical approval

The experimental procedures were approved by the Clinical Research Ethics Board at the University of Waterloo (ORE # 41774). The methods and protocols adhered to the recommendations outlined by the *Declaration of Helsinki* concerned with the use of human participants, except for registration in a database. All participants provided written informed consent.

### 2.2 Participants

Thirty‐two participants, *n* = 12 males, *n* = 10 females‐BC using monophasic birth control, and n = 10 females‐NBC, normally menstruating, participated in the study. The normally menstruating females‐NBC group was defined as having 8–14 cycles per year, and our participants had not missed any periods in the last year nor had they used any hormonal birth control in the past 12 months. The participants using hormonal birth control had to have been using their specific brand for at least 3 months, and for those using a method that was not an intrauterine device (IUD), they had to be consistent with their administration, within 2 h. Our females‐BC had all been using birth control for several years at the time of the study. Participants were between 18 and 33 years old, healthy, and met the current Canadian physical activity guidelines (Tremblay et al., 2011). Female participants were excluded if they were using a non‐hormonal intrauterine device or a progesterone only hormonal contraceptive, if they were pregnant, planning to be pregnant, or were breast feeding. Participants were excluded if they had a BMI >30 kg m^−2^, or had any cardiovascular, respiratory, or metabolic conditions that could impact their response to exercise. Participants abstained from alcohol, caffeine, and exhaustive exercise for >12 h prior to each testing visit.

### 2.3 Protocol overview

Testing occurred on 3 days over the course of approximately 3 weeks. On day 1 of testing, participants completed informed consent and health screening questionnaires followed by a maximal exercise test to volitional exhaustion on a cycle ergometer. Females‐BC completed day 1 on the placebo pills while females‐NBC completed day 1 during their menses or immediately after. Days 2 and 3 of testing each consisted of three separate 15–20‐min bouts of submaximal exercise at 30% of maximal work rate under three different conditions, which were randomized between participants but kept consistent between experimental days: room air, hypercapnia, or hyperoxia/hypoxia. Between each exercise bout, participants had a rest period of 5–10 min or until heart rate returned to resting levels. Day 2 took place 6 ± 6 days after the maximal exercise test, while day 3 took place 12 ± 4 days after day 2 (Table 1). Females‐BC completed day 2 and 3 during the active phase of their birth control while females‐NBC completed day 2 during their self‐reported low hormone phase (i.e., menses or shortly after in the follicular phase) and completed day 3 in the luteal phase after a positive ovulation test (Table 1).

**TABLE 1:** Participant demographics.

	Males (*n* = 12)	Females‐NBC (*n* = 10)	Females‐BC (*n* = 10)
Age (yrs)	24 ± 4	24 ± 3	22 ± 2
Height (cm)	178 ± 4	165 ± 7^a^, ^b^	172 ± 9^a^
Weight (kg)	77 ± 7	61 ± 15^a^	65 ± 7^a^
BMI (kg m^−2^)	24.2 ± 2.4	22.5 ± 4.1	21.9 ± 2.6
Hormonal IUD	N/A	N/A	6
Days between visit 1 and 2	6 ± 5	3 ± 2^b^	8 ± 7
Days between visit 2 and 3	14 ± 5	12 ± 4	11 ± 4
Days between + ovulation test and day 3	n/a	1.8 ± 1	n/a

*Note*: Mean ± SD.

Abbreviations: BC, using hormonal birth control; BMI, body mass index; IUD, intrauterine device; NBC, not using hormonal birth control.

^a^ Significant difference from males, *p <* 0.05.

^b^ Significant difference from females‐BC, *p* < 0.05.

### 2.4 Day 1—Maximal exercise testing

The maximal exercise test was conducted on a cycle ergometer (Monark LC7TT, Vansbro, Sweden), with the seat and handlebar position recorded and kept consistent for all subsequent sessions within participants. After a 5–10‐min rest period while sitting on the bike, participants completed a maximal exercise test in which females and males started at 40 W and 60 W, respectively, and increased by 20 W per minute until volitional exhaustion. Participants always breathed room air during maximal exercise testing.

### 2.5 Days 2 and 3—Submaximal exercise testing

**FIGURE 1:**
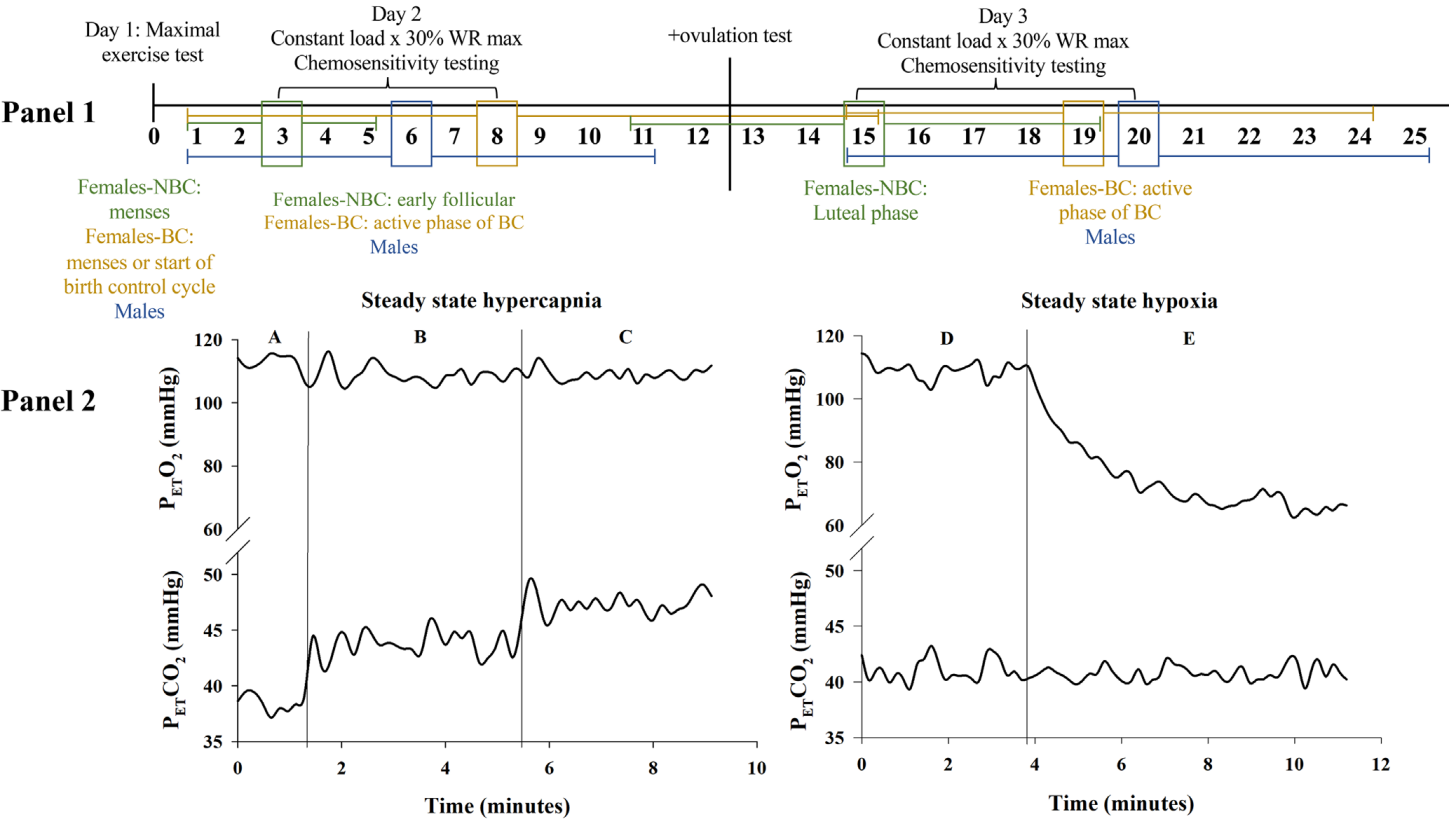
A protocol schematic (panel 1) and raw trace of the steady state hypercapnic and hypoxic protocols (panel 2). The protocol schematic shows the number of days following the maximal exercise test that day 2 and 3 were completed for males, females not using hormonal birth control (NBC), and females using hormonal birth control (BC). The boxes show the mean for each group (color indicated), while the bars show the standard deviation. In the raw trace (panel 2), (A) the pre‐stimulus end‐tidal CO_2_ (P_ET_CO_2_) and end‐tidal O_2_ (P_ET_CO_2_) used to determine the steady state values for the increased CO_2_ as well as the iso‐oxic value. (B) the +4 mmHg step change in P_ET_CO_2_ with constant P_ET_O_2_. (C) the +7 mmHg step change in P_ET_CO_2_ with constant P_ET_O_2_. (D) the pre‐hypoxic P_ET_CO_2_ used to determine the iso‐capnic value for hypoxia. (E) the decrease in P_ET_O_2_ followed by several minutes at steady state.

Testing days 2 and 3 were identical with three, 15–20‐min bouts of continuous submaximal exercise, each separated by 5–10 min of rest during which participants were disconnected from the mouthpiece. The exercise was completed at 30% maximal work rate derived from the maximal exercise test. Each bout of exercise was in one of the following conditions: room air, hypercapnia, or hyperoxia/hypoxia. The room air bout had no inspired air manipulations while the hypercapnia and hyperoxic/hypoxia manipulations are described below. After completing day 2 of testing, the NBC participants received an ovulation test kit (Clearblue® Advanced Digital Ovulation Test, SPD Swiss Precision Diagnostics GmbH, Geneva, Switzerland) and were instructed to use one test at approximately the same time each day, until they received an indicator of ovulation, at which time they completed day 3 of testing. All females‐NBC received a positive ovulation test. The females‐NBC performed day 3 within 1.8 ± 1.0 days of the positive ovulation test. Females‐BC completed days 2 and 3 of testing in the same cycle while in the active phase of their hormonal contraceptive. Males and females‐BC completed day 3 between 7 and 20 days after day 2 (Figure 1). The variable time between day 2 and 3 for females‐BC females and males was used to mimic the range for females‐NBC from menses to ovulation, providing a similar spread of testing days.

### 2.6 Hypercapnic condition

Once steady state exercise was reached, confirmed visually by no further increase in heart rate and ventilation, the transient hypercapnic tests were performed. Each test consisted of two breaths of a hypercapnic gas mixture (10% CO_2_; ~21% O_2_) repeated five times, each separated by 45–60 s (Mann et al., 2022; Wright et al., 2023). The hypercapnic breaths were given by switching the participants from room air to a Douglas bag using a three‐way valve that provided no audible or visual cue to alert the participant. Following the five transient tests, the participant continued exercising to allow steady state to resume for 1–2 min, at which time pre‐stimulus end‐tidal CO_2_ and O_2_ (P_ET_CO_2_ and P_ET_O_2_, respectively) were recorded for the subsequent steady state hypercapnic tests. An end‐tidal forcing system, described elsewhere (Tymko et al., 2015), was then used to increase P_ET_CO_2_ to +4 mmHg and then +7 mmHg from the pre‐stimulus values. Each step increase in P_ET_CO_2_ was held for 4 min in duration. Participants were kept iso‐oxic with P_ET_O_2_ held constant at the pre‐stimulus value for the entire duration of the steady state CO_2_ period, (Figure 1). Following the +7 mmHg step, the participant was switched back to room air for a final 2 min of exercise while mixed expired gases were collected to calculate oxygen uptake (V̇O_2_).

### 2.7 Hyperoxic/hypoxic condition

Transient hyperoxic tests were performed once steady state was reached, which consisted of 2 breaths of 100% O_2_ repeated 3–5 times, separated by 60–90 s of room air. The breaths were given from a Douglas bag connected to a valve that allowed the participant to be switched from room air to 100% O_2_ without an audible or visual cue. After the transient hyperoxic tests were completed, the participant continued cycling for 1–2 min until steady state was achieved and P_ET_CO_2_ and P_ET_O_2_ were recorded. Participants were then brought to an oxyhaemoglobin saturation of 80%–82%, over ~3 min, and kept there for 4–5 min of steady state hypoxic exercise, while P_ET_CO_2_ was held at the pre‐stimulus value (i.e., isocapnia), using an end‐tidal forcing system (Figure 1). While P_ET_O_2_ can provide a reasonable estimation of arterial PO_2_ during rest, there is an increase in the alveolar‐to‐arterial oxygen gradient with exercise that is highly variable between participants (Stickland et al., 2013). As such, using end‐tidal forcing to lower each participant's P_ET_O_2_ to a specific value (e.g., 55 mmHg) would provide a highly variable arterial PO_2_ between participants. Thus, for our hypoxic stimulus, we held each participant at an oxyhaemoglobin saturation (SpO_2_) of 80%–82%, rather than a specific P_ET_O_2_, as this would allow for a more consistent arterial PO_2_ to stimulate the peripheral chemoreceptors. Following the hypoxia stimulus, participants were switched to room air for the final 2 min, where mixed expired gases were collected.

### 2.8 Data collection

A 16‐channel analog‐to‐digital data acquisition system (Powerlab/16sp model ML 795; ADInstruments, Colorado Springs, CO) was used to record raw data at 200 Hz. Mixed expired gases were measured continuously during the maximal exercise test. During the submaximal exercise trials, end‐tidal gases were measured continuously at the mouth until the last 2 min at which point the sample line was switched to the mixing chamber to measure mixed expired gases using calibrated O_2_ and CO_2_ analyzers (AEI Technologies S‐3‐A/I and CD‐3Am, respectively; Applied Electrochemistry, Bastrop, TX). Heart rate was assessed using a polar heart rate monitor (Model T34; Polar Electro Oy, Kempele, Finland). One pulse oximeter (Nonin 7500; Nonin Medical Inc., Plymouth, Minn., USA) on the finger was used to measure SpO_2_ during the maximal exercise test and a second (Model M170; Shenzhen Fitfaith Technology Co. Ltd., Shenzhen, China) was used during the submaximal exercise. During the maximal exercise test, two separate pneumotachometers (model 3813; Hans Rudolph, Kansas City, MO) were used to measure inspired and expired flow. The expired pneumotachometer was heated to 37°C, while the inspired was kept at room temperature. The pneumotachometers were independently calibrated using room air and a 3 L syringe. A two‐way nonrebreathing valve (model 2700; Hans Rudolph, Kansas City, MO) was connected to the participant using a face mask (model 7450 V2, Hans Rudolph, Kansas City, MO) and to the pneumotachometers using large bore tubing, with a mixing chamber distal to the expired pneumotach to measure mixed expired gases.

During the submaximal exercise on days 2 and 3, a single pneumotachometer heated to 37°C was used to measure both inspired and expired flow. A modified sequential gas delivery circuit was used to improve end‐tidal gas control (Farra et al., 2016).

### 2.9 Data analysis

Maximal exercise variables were determined from a 30 s average taken at the end of the test. For each of the three conditions (room air, hypercapnia, and hypoxia/hyperoxia), the last 30 s of exercise while breathing room air was averaged for the trial to determine oxygen consumption (V̇O_2_) and carbon dioxide output (V̇CO_2_) when the sample line was connected to the mixing chamber. All other variables for the room air trial were determined as a 1‐min average taken at the end of exercise, while the sample line was connected at the mouth prior to when the sample line was transferred to the mixing chamber to measure mixed expired gases. The hypercapnic data was analyzed in two parts: transient and steady state responses. The transient hypercapnic response was determined as the quotient between the change in inspired ventilation (ΔV̇_I_) and ΔP_ET_CO_2_, with the deltas calculated as the difference between the peak and the respective pre‐stimulus averages, as described previously (Mann et al., 2022). The response was scaled for body surface area. Only 9 of 10 females‐NBC were included in the transient hypercapnic analysis because one participant would consistently cough after the CO_2_ breaths rendering analysis not possible.

For steady state hypercapnia, the V̇_I_ delta was taken between the last‐minute average of baseline and the P_ET_CO_2_ of +4 and +7, and the change in V̇_I_ across all P_ET_CO_2_ was used to determine the slope of the response. The transient hyperoxic response was the delta between the pre‐stimulus V̇_I_ and the average of the two lowest V̇_I_ taken while the P_ET_O_2_ was still elevated (>130 mmHg). Each participant had 3–5 responses that were analyzed separately, and then combined for an average response for each participant. Finally, the hypoxic response was taken as the averaged last minute when SpO_2_ was lowered to ~80% where the ventilatory response was the percent change between baseline and 80% SpO_2_ per percent desaturation. The duration of each condition was determined as the total time spent cycling, expressed in seconds, and did not include any rest prior to the onset of exercise.

### 2.10 Statistical analysis

Statistical analyses were completed using SigmaPlot v15. The descriptive and maximal exercise variables, as well as the submaximal work rate, were analyzed with a one‐way ANOVA to compare all three groups: males, females‐NBC, and females‐BC.

The steady state CO_2_ response at +4 mmHg and +7 mmHg was analyzed with a two (day 2 and day 3) by three (male, females‐NBC, and females‐BC) repeated measures ANOVA. The change in CO_2_ for each step, and V̇_I_ prior to the CO_2_ stimulus were also analyzed with the same two by three ANOVA design. The additional data for the steady state CO_2_ trials (i.e., delta heart rate, SpO_2_, and P_ET_O_2_) were analyzed with a one‐way ANOVA (male, females‐NBC, and females‐BC) for each step and day.

For the hypoxia trials, the hypoxic ventilatory response, P_ET_CO_2_, and the pre‐stimulus V̇_I_ were analyzed with the same day by group two‐way repeated measures ANOVA design. The change in HR and SpO_2_ were analyzed with a one‐way ANOVA for day 2 and day 3. For the hyperoxic response, the V̇_I_ percent decrease and pre‐stimulus V̇_I_ were analyzed with the same day by group two‐way repeated measures ANOVA.

Any significant F ratios were analyzed with a Tukey's post hoc test. Data that did not approximate a normal distribution were analyzed with the Kruskal–Wallis ANOVA on ranks, with the Dunn's method for multiple comparisons. For each condition, the within group relationship between day 2 and 3 was analyzed with a regression. Intraclass correlation coefficient (ICC) tests were conducted in R version 4.4.0 for the day 2 versus day 3 responses to transient and steady state hypercapnia, hyperoxia, and hypoxia.

## 3 RESULTS

### 3.1 Demographics and maximal exercise

Participant demographics are shown in Table 1, and the maximal exercise data are shown in Table 2. The male participants had a greater mass and height compared to the female groups, with no difference between the females‐NBC and BC groups, except for females‐BC being taller (Table 1). Males had significantly greater work rates and V̇O_2_ than both female groups, while the females‐NBC had a significantly lower heart rate than both the females‐BC and males at maximal exercise (Table 2). There was no significant difference in respiratory exchange ratio or ventilatory equivalents between groups, suggesting that they all achieved maximal exercise to the same degree, despite the differences in size and work rate.

**TABLE 2:** Day 1 end exercise data.

	Males (*N* = 12)	Females‐NBC (*N* = 10)	Females‐BC (*N* = 10)
Work rate (W)	308 ± 48	192 ± 64^a^	214 ± 35^a^
Heart rate (bpm)	190 ± 6	180 ± 4^a^, ^b^	190 ± 7
SpO_2_ (%)	94 ± 5	96 ± 4	95 ± 4
V̇_E_ (L min^−1^)	177 ± 28	103 ± 27^a^	114 ± 18^a^
V_T_ (L)	3.2 ± 0.4	2.0 ± 0.4	2.2 ± 0.3
Fb (breaths min^−1^	56 ± 14	52 ± 8	53 ± 12
V̇O_2_ (L min^−1^)	4.2 ± 0.6	2.5 ± 0.8^a^	2.8 ± 0.4^a^
V̇O_2_ (mL kg^−1^ min^−1^)	55.1 ± 9.7	39.6 ± 7.3^a^	43.1 ± 3.5^a^
V̇CO_2_ (L min^−1^)	4.8 ± 0.6	2.8 ± 0.7^a^	3.2 ± 0.5^a^
V̇_E_/V̇O_2_	43 ± 5	44 ± 8	41 ± 3
V̇_E_/V̇CO_2_	37 ± 4	37 ± 4	36 ± 4
RER	1.15 ± 0.08	1.17 ± 0.14	1.13 ± 0.08

*Note*: Data presented as mean ± SD.

Abbreviations: BC, using hormonal birth control; Fb, breathing frequency; NBC, not using hormonal birth control; RER, respiratory exchange ratio; SpO_2_, oxygen saturation from pulse oximetry; V̇CO_2_, carbon dioxide production; V̇_E_, ventilation; V̇O_2_, oxygen uptake; V_T_, tidal volume.

^a^ Significant difference from males, *p <* 0.05.

^b^ Significant difference from females‐BC, *p* < 0.05.

### 3.2 Room air submaximal exercise

Table 3 presents data from the room air submaximal exercise condition. Except for exercise duration (+41 s), there were no differences between days 2 and 3. Males had a higher absolute work rate for the submaximal conditions due to a higher work rate achieved during the maximal exercise test, and subsequently they had a greater V̇O_2_, V̇CO_2_, and V̇_I_ than both female groups. Despite the same relative work rate, females‐BC had a higher heart rate for the duration of the room air exercise compared to males. There were no differences between the females‐NBC and the females‐BC groups.

**TABLE 3:** Cardiorespiratory variables during the final minute of the room air trials.

	Day 2 (all participants)	Day 3 (all participants)	Males	Females‐NBC	Females‐BC	*p* Value		
						Group	Day	Interaction
Participants (*n*)	32	32	12	10	10			
Work rate (W)	73 ± 21	73 ± 21	92 ± 14	58 ± 20^a^	65 ± 11^a^	**<0.001**	n/a	n/a
Heart rate (bpm)	129 ± 15	127 ± 12	121 ± 9.8	130 ± 11	135 ± 16^a^	**0.030**	0.197	0.892
SpO_2_ (%)	97 ± 2	97 ± 2	97 ± 2	97 ± 2	96 ± 1	0.558	0.117	0.376
P_ET_O_2_ (mmHg)	109 ± 6	108 ± 5	106 ± 6	110 ± 4	109 ± 5	0.057	0.289	0.563
P_ET_CO_2_ (mmHg)	43 ± 3	43 ± 4	45 ± 3	42 ± 3^a^	42 ± 4^a^	**0.029**	0.170	0.614
V̇_I_ (L min^−1^)	47 ± 8	46 ± 7	51 ± 7	45 ± 9^a^	44 ± 6^a^	**0.032**	0.432	0.524
V̇O_2_ (L min^−1^)	1.54 ± 0.4	1.53 ± 0.4	1.88 ± 0.2	1.31 ± 0.3^a^	1.36 ± 0.2^a^	**<0.001**	0.713	0.758
V̇CO_2_ (L min^−1^)	1.27 ± 0.3	1.28 ± 0.3	1.57 ± 0.2	1.10 ± 0.3^a^	1.11 ± 0.2^a^	**<0.001**	0.826	0.825
RER	0.83 ± 0.05	0.84 ± 0.03	0.84 ± 0.04	0.84 ± 0.04	0.82 ± 0.04	0.322	0.200	0.721
Exercise duration (s)	1048 ± 160	1089 ± 132^b^	1052.1 ± 155.0	1045.1 ± 154.4	1110.0 ± 127.1	0.529	**0.019**	0.548

*Note*: Values are mean ± SD.

Abbreviations: BC, using hormonal birth control; NBC, not using hormonal birth control; P_ET_CO_2_, end‐tidal carbon dioxide pressure; P_ET_O_2_, end‐tidal oxygen pressure; RER, respiratory exchange ratio; SpO_2_, oxygen saturation from pulse oximetry; V̇CO_2_, carbon dioxide production; V̇_E_, ventilation; V̇O_2_, oxygen uptake.

^a^ Significantly different from males.

^b^ Significantly different from Day 2.

### 3.3 Transient hypercapnia

**FIGURE 2:**
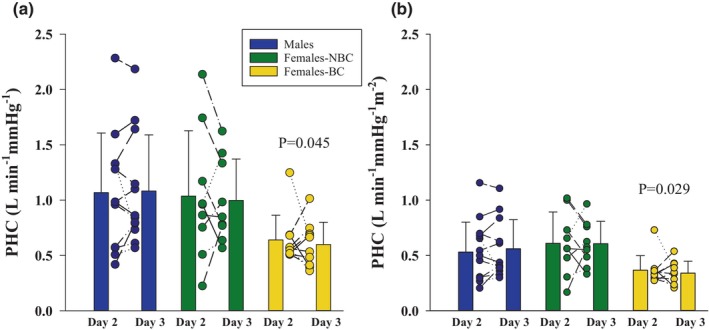
Peripheral hypercapnic chemoresponse (PHC) for each group on day 2 (follicular phase) and day 3 (luteal phase). (a) The PHC in males, females not using hormonal birth control (NBC), and females using hormonal birth control (BC), the *p* value indicates a significant difference to males. (b) The PHC for each group corrected for body surface area where the *p* value indicates a significant difference to females‐NBC. The females‐NBC response was determined with nine participants. There was no effect of day for any group.

**FIGURE 3:**
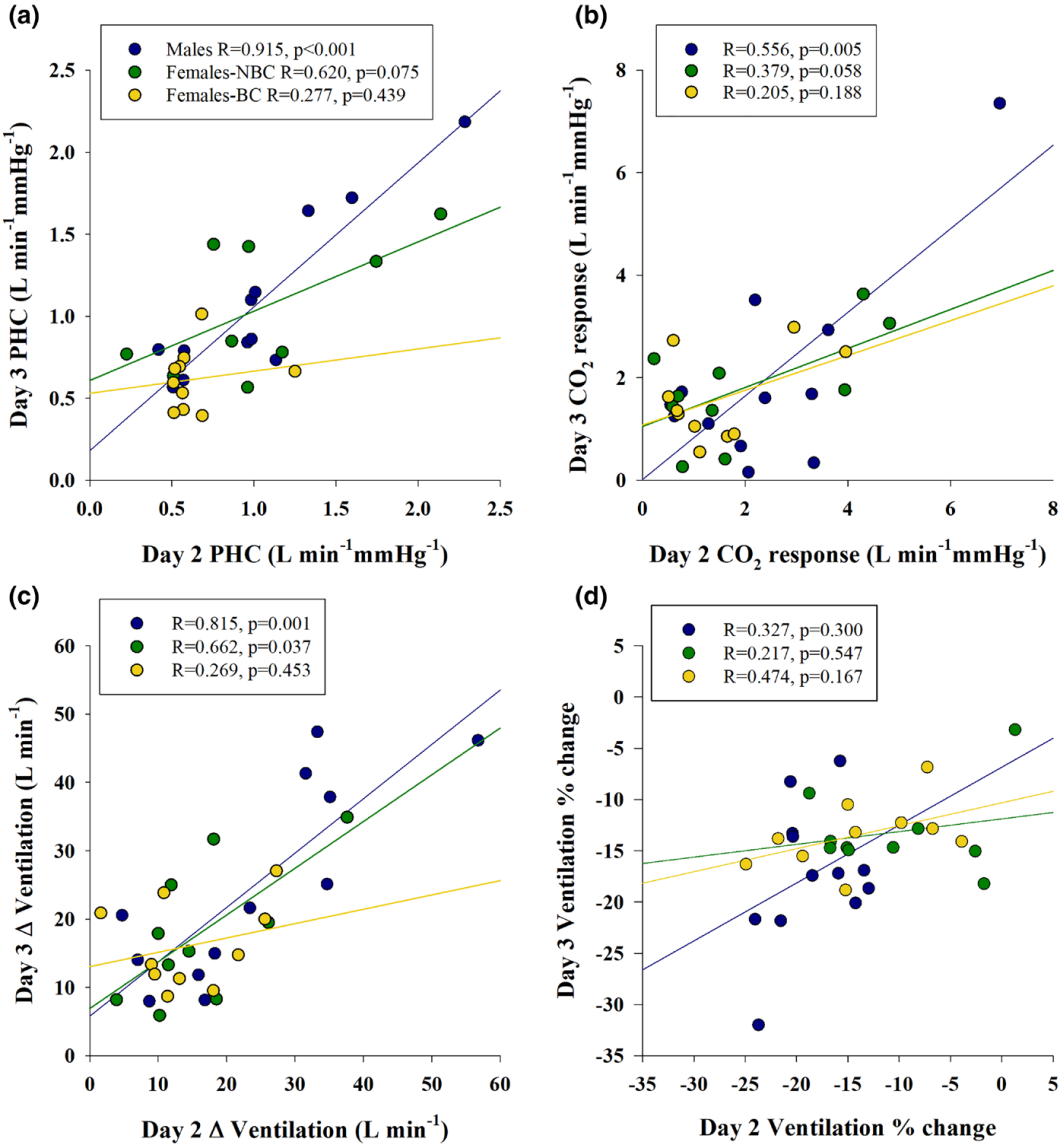
The relationship between day 2 and day 3 for each chemoresponse trial. Each graph includes the data for males, females not using hormonal birth control (NBC) and females using hormonal birth control (BC) for day 2, the follicular phase, and day 3, the luteal phase for females‐NBC. (a) The peripheral hypercapnic chemoresponse (PHC) relationship between day 2 and 3 for males, females‐NBC, and females‐BC. Males had a significant strong relationship between the response on day 2 and 3 while both female groups had no significant relationship. (b) the relationship of the steady state hypercapnic response, the slope of ventilation and end‐tidal CO_2_ (P_ET_CO_2_) at pre, +4 mmHg, and +7 mmHg, between day 2 and 3. Males had a significant relationship, both female groups had a nonsignificant relationship, however, females‐NBC were trending towards significance. (c) The relationship on day 2 and 3 for the change in ventilation during steady state hypoxia. Both males and females‐NBC have a significant relationship, while females‐BC did not have a significant relationship. (d) The relationship between day 2 and day 3 for the transient hyperoxic response. There were no significant relationships between males, females‐NBC, and females‐BC.

The peripheral hypercapnic response (PHC) to transient CO_2_ is shown in Figure 2 as individual data points as well as the average for each group for day 2 and day 3. There was no difference (*p* > 0.05) between days within any group. The females‐BC had a lower response than males (*p* = 0.045) (panel A). The difference between female BC and males did not persist when scaled for body surface area; however, there was a difference between females‐BC and females‐NBC (*p* = 0.029) (panel B). The pre‐stimulus V̇_I_ and P_ET_CO_2_ were not different on both day 2 and 3 for all groups; however, the males had a significantly higher pre‐V̇_I_ than females‐NBC and females‐BC on day 2 (Table 4). There was no difference (*p* = 0.154) in the pre‐stimulus V̇_I_ between day 2 and 3 within any group. When the PHC was examined between day 2 and day 3, males had a significant strong relationship while both female groups had no statistically significant relationship (Figure 3a). The ICC for the PHC response between day 2 and 3 were 0.94, excellent reliability, for males (*p* < 0.01), 0.82, good reliability, for females‐NBC (*p* = 0.013), and 0.34 for females‐BC (*p* = 0.27).

**TABLE 4:** Transient hypercapnia data.

	Males (*n* = 12)		Females‐NBC (*n* = 9)		Females‐BC (*n* = 10)		Group	*p* Value	Interaction
	Day 2	Day 3	Day 2	Day 3	Day 2	Day 3		Day	
Pre V̇_I_ (L min^−1^)^a^	49 ± 6	47 ± 6	43 ± 7	41 ± 11	41 ± 4	41 ± 3	**0.016**	0.170	0.568
Pre P_ET_CO_2_ (mmHg)^b^	46 ± 3	46 ± 3	42 ± 2	43 ± 3	43 ± 4	44 ± 4	**0.027**	0.80	0.509
Coefficient of Variation (%)	39 ± 27	37 ± 15	39 ± 12	35 ± 12	41 ± 12	38 ± 16	0.610	0.352	0.802
Time to peak V̇_I_ (s)	8.7 ± 2.2	9.5 ± 2.3	8.0 ± 2.0	8.2 ± 1.1	9.2 ± 0.9	9.0 ± 0.9	0.178	0.553	0.426

*Note*: Data are means ± SD. Day 2 took place during the follicular phase, while day 3 was during the luteal phase for females‐NBC.

Abbreviations: BC, using hormonal birth control; NBC, not using hormonal birth control; P_ET_CO_2_, end‐tidal carbon dioxide; V̇_I_, inspired ventilation.

^a^ Indicates a significant difference between males and females‐BC.

^b^ Indicates a significant difference between males and females‐NBC.

### 3.4 Steady state hypercapnia

**FIGURE 4:**
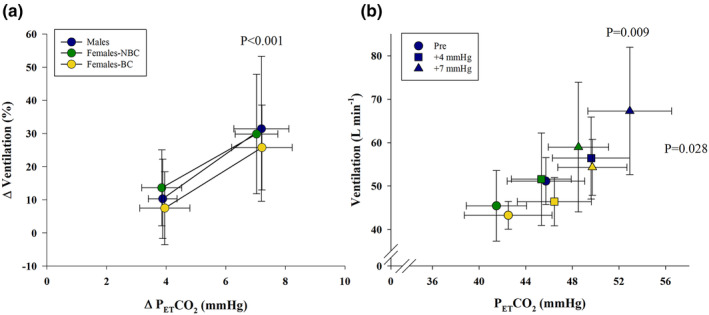
The average steady state hypercapnic response for males, females not using hormonal birth control (NBC), and females using hormonal birth control (BC). (a) The change in ventilation for the change in end‐tidal CO_2_ (P_ET_CO_2_) for each steady state step. The *p* value indicates a significant increase in ventilation for each group with no difference between each group. (b) The absolute ventilation and P_ET_CO_2_ for each group at pre, +4 mmHg, and +7 mmHg. Each point in both graphs is an average of day 2 and 3. Males had a significantly higher ventilation than females‐BC (*p* = 0.028) and P_ET_CO_2_ than females‐NBC (*p* = 0.009) with no effect of the steady state step. Ventilation and P_ET_CO_2_ was higher at each step consecutively, *p* < 0.001.

**FIGURE 5:**
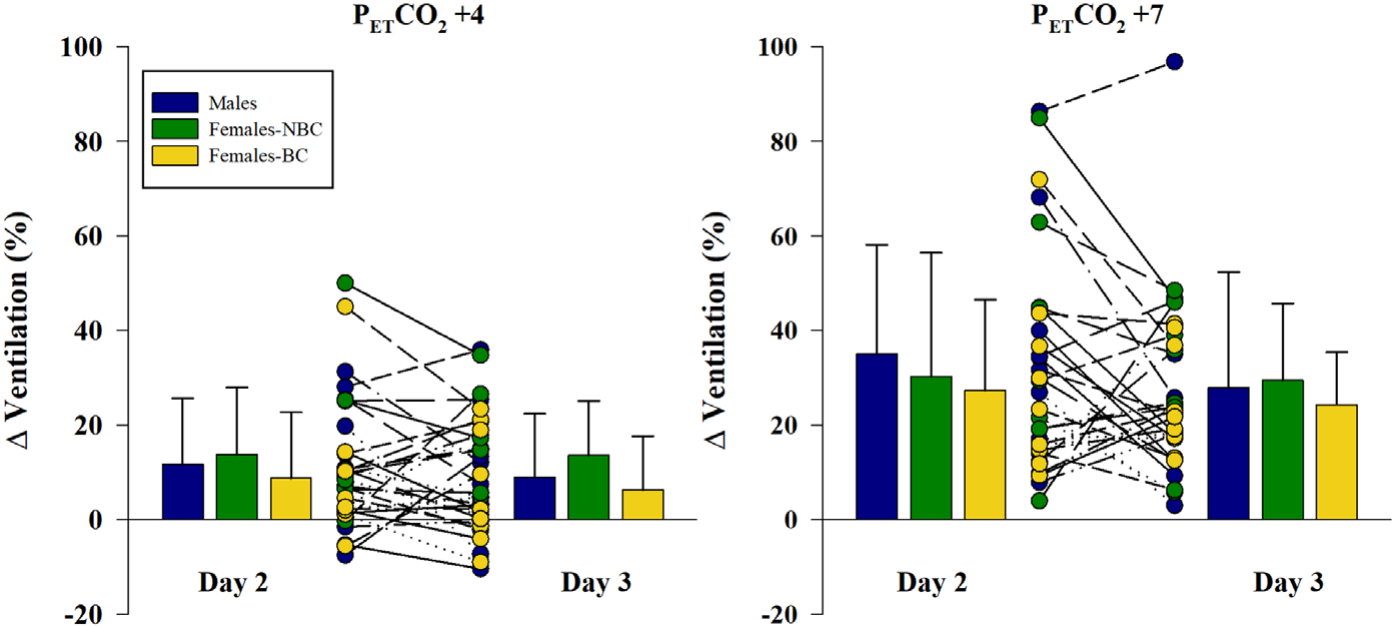
The average and individual response to each step of steady state hypercapnia females not using hormonal birth control (NBC), and females using hormonal birth control (BC). Day 2 and 3 are compared with no significant differences in the change in ventilation at the steady state end‐tidal CO_2_ (P_ET_CO_2_) of +4 mmHg (left) and +7 mmHg (right) for either day or any group. Day 2 took place during the follicular phase, while day 3 was during the luteal phase for females‐NBC.

The increase in P_ET_CO_2_ for the steady state hypercapnic tests was not different between groups (Figure 4). Steady state hypercapnia caused an increase in ventilation, with a P_ET_CO_2_ of +7 mmHg causing a significantly greater increase than +4 mmHg (Figure 4). However, there was no significant difference in the ventilatory response between days or groups for either step of P_ET_CO_2_ (Figure 5). Additionally, P_ET_O_2_ changed minimally from the pre‐stimulus value, with deltas ranging from −3.2 to 2.3 mmHg and minimal changes in SpO_2_ or heart rate during the steady state hypercapnia (Table 5). The slope of the ventilatory response to increased P_ET_CO_2_ was not significantly different between groups or days, (males 2.4 ± 1.8 vs. 2.0 ± 1.9; females‐NBC 2.0 ± 1.7 vs. 1.8 ± 1.1; and females‐BC 1.5 ± 1.1 vs. 1.6 ± 0.9 L min^−1^ mmHg^−1^, each for day 2 and 3, respectively). The hypercapnic ventilatory slopes between day 2 and 3 within each group indicated a significant weak relationship within males, but no significant relationship for females‐NBC or females‐BC (Figure 3b). ICC results for the ventilatory slopes were significant for males, good reliability, 0.85 (*p* < 0.01) and females‐NBC, moderate reliability, 0.73 (*p* = 0.031) but not for females‐BC 0.64 (*p* = 0.071).

**TABLE 5:** Steady state hypercapnic stimulus data.

+4	Males (*n* = 12)		Females‐NBC (*n* = 10)		Females‐BC (*n* = 10)		*p* Values		
	Day 2	Day 3	Day 2	Day 3	Day 2	Day 3	Group	Day	Interaction
HR Δ (bpm)	−1 ± 5	1 ± 2	3 ± 3^a^	2 ± 2	1 ± 2	1 ± 6	**0.029**	0.935	n/a
P_ET_O_2_ Δ (mmHg)	−1.5 ± 4.5	−2.1 ± 2.4	1.5 ± 9.5	−3.2 ± 2.5	−1.1 ± 2.6	−1.1 ± 2.7	0.402, 0.273	0.127	n/a
SpO_2_ Δ (%)	−0.3 ± 0.7	−0.8 ± 0.9	−0.3 ± 0.5	−0.3 ± 0.8	−1 ± 1.2	−0.3 ± 1.0	0.153, 0.289	0.988	n/a
Pre V̇_I_ (L min^−1^)^b^	51 ± 6	52 ± 6	45 ± 7	46 ± 10	43 ± 4	44 ± 4	**0.011**	0.665	0.957
Pre P_ET_CO_2_ (mmHg)	47 ± 3^c^	45 ± 4	41 ± 3^a^	42 ± 3	42 ± 4^a^	43 ± 4	**0.012**	0.660	**0.003**

+7	Males		Females‐NBC		Females‐BC				
	Day 2	Day 3	Day 2	Day 3	Day 2	Day 3			
HR Δ (bpm)	2 ± 4	3 ± 4	5 ± 4	2 ± 3	3 ± 3	2 ± 6	0.164, 0.671	0.324	n/a
P_ET_O_2_ Δ (mmHg)	−0.5 ± 4.7	−1.2 ± 3.0	2.6 ± 5.0	−1.2 ± 1.9	0.1 ± 3.7	−0.5 ± 3.0	0.532	0.78	0.334
SpO_2_ Δ (%)	−0.5 ± 0.9	−0.7 ± 0.9	−0.4 ± 1.6	−0.8 ± 0.8	−0.5 ± 1.6	−0.2 ± 1.4	0.633, 0.799	0.303	n/a

*Note*: Data are means ± SD. Where there are two *p* values the first reflects the comparison for day 2, and the second reflects day 3. Day 2 took place during the follicular phase, while day 3 was during the luteal phase for females‐NBC.

Abbreviations: BC, using hormonal birth control; HR Δ, change in heart rate from baseline; NBC, not using hormonal birth control; P_ET_CO_2_, end‐tidal carbon dioxide; P_ET_O_2_ Δ, change in end‐tidal oxygen from baseline; SpO_2_ Δ, change in saturation from pulse oximetry from baseline; V̇_I_, inspired ventilation.

^a^ Indicates a significant difference to males for the same day.

^b^ Indicates a significant difference between males and females‐BC.

^c^ Indicates a significant difference between days.

### 3.5 Transient hyperoxia

**FIGURE 6:**
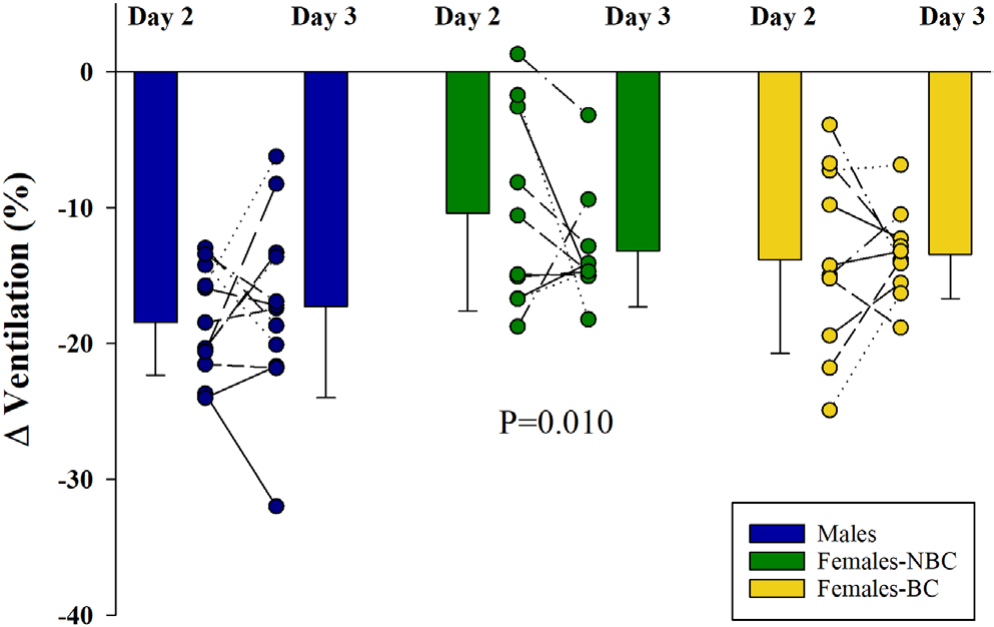
The decrease in ventilation in response to transient hyperoxia. The average and individual response to 100% oxygen on day 2 and day 3 for males, females not using hormonal birth control (NBC), and females using hormonal birth control (BC). The females‐NBC had a significantly smaller change in ventilation compared to the male participants, *p* = 0.010. Day 2 took place during the follicular phase, while day 3 was during the luteal phase for females‐NBC.

The transient hyperoxic response is shown in Figure 6. The females‐NBC had a lower response (*p* = 0.010) compared to the male participants, with no effect of day or interaction between groups. The females‐BC had a lower ventilation (average of day 2 and 3 because of no significant difference between days) (39.3 ± 6.0 L min^−1^) compared to males (46.1 ± 4.3 L min^−1^) (*p* = 0.011) prior to the hyperoxic stimulus; however, no differences existed in the response compared to the males or females‐NBC (40.7 ± 6.5 L min^−1^) (*p* > 0.05). There was no statistically significant relationship between the response on day 2 and day 3 between the groups (Figure 3d). The ICC for the hyperoxic response between day 2 and 3 were 0.47 for males, 0.32 for females‐NBC, and 0.58 for females‐BC, *p* > 0.05 for all groups.

### 3.6 Steady state hypoxia

**FIGURE 7:**
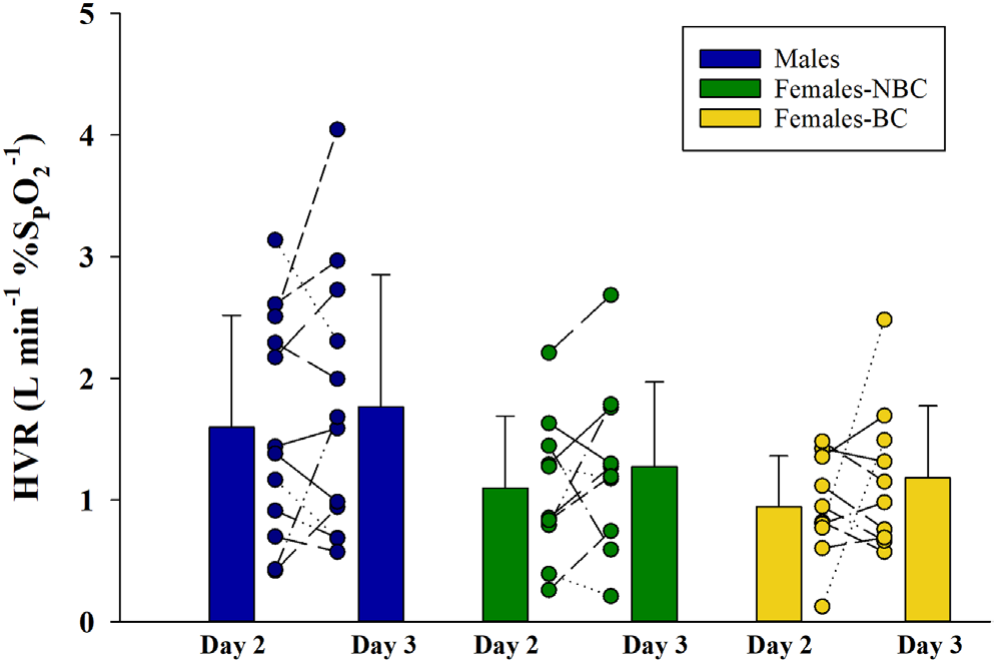
The hypoxic ventilatory response. The response of males, females not using hormonal birth control (NBC) and females using hormonal birth control (BC) to steady state hypoxia. There were no significant differences between days or groups, *p* > 0.05. HVR, hypoxic ventilatory response; SpO_2_, oxygen saturation from pulse oximetry.

Steady state hypoxia is shown in Figure 7 and Table 6. The P_ET_CO_2_ was held constant during the hypoxic periods with a change from pre‐hypoxia of less than 0.5 mmHg (Table 6). Similarly, SpO_2_ and heart rate were not different between groups during hypoxic exercise (Table 6). Steady state hypoxia caused no difference in the increase in ventilation between groups (*p* = 0.101) or days (*p* = 0.114) (Figure 7). The females‐BC had a lower ventilation prior to the hypoxic stimulus (Table 6); however, their response was proportionate to the other groups. Lastly, males had a strong positive relationship between the change in V̇_I_ on day 2 and day 3, females‐NBC had a moderate positive relationship, and females‐BC had no significant relationship, Figure 3c. The responses between day 2 and 3 had a significant ICC for males 0.91, excellent reliability, and females‐NBC 0.81, good reliability, (*p* < 0.01 and 0.011, respectively) and not significant for females‐BC, 0.46 (*p* = 0.19).

**TABLE 6:** Steady state hypoxic data.

	Males (*n* = 12)		Females‐NBC (*n* = 10)		Females‐BC (*n* = 10)		*p* Value
	Day 2	Day 3	Day 2	Day 3	Day 2	Day 3	
HR Δ bpm	14 ± 5	15 ± 5	16 ± 9	16 ± 6	18 ± 8	20 ± 6	0.485, 0.167
P_ET_CO_2_ Δ mmHg	0.3 ± 1.2	−0.3 ± 1.2	0.3 ± 1.1	0.4 ± 0.7	0.4 ± 1.4	0.3 ± 1.1	0.440
SpO_2_ Δ %	−15 ± 2	−14 ± 2	−15 ± 5	−15 ± 5	−15 ± 3	−14 ± 3	0.876, 0.943
Pre V̇_I_ (L min^−1^)^a^	50 ± 5	48 ± 6	45 ± 7	43 ± 9	42 ± 7	42 ± 7	**0.020**
V̇_I_ Δ (L min^−1^)	24 ± 15	25 ± 15	16 ± 10	18 ± 10	15 ± 8	16 ± 7	0.127

*Note*: Data are means ± SD. *p* Values are for the effect of group, as there was no difference between days. Where there are two *p* values the first reflects the comparison for day 2, and the second reflects day 3. Day 2 took place during the follicular phase, while day 3 was during the luteal phase for females‐NBC.

Abbreviations: BC, using hormonal birth control; HR Δ, change in heart rate from baseline; NBC, not using hormonal birth control; P_ET_CO_2_, end‐tidal carbon dioxide; SpO_2_ Δ, change in saturation from pulse oximetry from baseline; V̇_I_, inspired ventilation.

^a^ Indicates a significant difference between males and females‐BC.

### 3.7 Variability of transient chemoreflex tests

The within day variability was analyzed using the coefficient of variation for the PHC and the hyperoxic response. The within‐day coefficient of variation for the PHC showed no difference between days or groups (Table 4). The hyperoxic response had lower coefficients of variation (males 9.2 ± 4.9% and 8.0 ± 3.0%; females‐NBC 11.3 ± 9.2% and 11.7 ± 3.9%; and females‐BC 6.3 ± 3.4% and 8.4 ± 3.8%, day 2 and 3 for each pair, respectively) than PHC (Table 4). Females‐BC had a lower hyperoxic coefficient of variation compared to females‐NBC (*p* = 0.046) but not males (*p* = 0.717). The between‐day coefficient of variation for the PHC and hyperoxia showed no significant difference between groups (PHC: males 14.9 ± 13.9%, females‐NBC 27.0 ± 21.3%, females‐BC 21.3 ± 11.9%, and hyperoxia: males 4.6 ± 3.2%, females‐NBC 4.5 ± 4.3%, and females‐BC 4.0 ± 2.7% *p* > 0.05). Similarly, there was no difference in the between‐day coefficient of variation for steady state hypercapnia (males 50.6 ± 37.7%, females‐NBC 50.1 ± 35.2%, females‐BC 43.0 ± 27.6%) and hypoxia (males 26.4 ± 24.4%, females‐NBC 31.1 ± 19.4%, and females‐BC 33.6 ± 34.4%, *p* > 0.05).

## 4 DISCUSSION

### 4.1 Major findings

There were two main findings from this study. First, there was no effect of test day for any of our measures of ventilatory sensitivity in males, females‐NBC, or females‐BC. The lack of effect between the two measured days suggests that there is a large day‐to‐day biological variability in ventilatory sensitive and/or greater ventilatory control inputs during submaximal aerobic exercise. However, the female BC participants had less reproducible data than the males and females‐NBC, suggesting that hormonal contraceptive use does not cause a consistent effect on the exercise chemoresponses. Second, regardless of test day, the males had greater transient hypercapnic and hyperoxic ventilatory responses than females. For normally menstruating females (NBC), there was no effect of menstrual cycle phase on the measured parameters. Overall, our results suggest that the menstrual cycle has minimal and inconsistent impacts on chemosensitivity during acute submaximal aerobic exercise.

### 4.2 Variations in chemosensitivity

The normal biological variation in hormone concentration across the menstrual cycle or within females using hormonal contraceptives likely does not impact the chemoreceptors response greater than the anticipated “day‐to‐day” variability. We base this assertion on the similar coefficient of variation for the PHC, both between‐ and within‐days, and the within‐day coefficient of variation for the hyperoxia, steady state iso‐oxic hypercapnia, and isocapnic hypoxia ventilatory response, as well as no significantly different response between day 2 and day 3 for all groups. The excellent and good reliability for males and females‐NBC between day 2 and 3 for the PHC, steady state hypercapnia, and hypoxia also suggests that the chemoresponse during exercise is not affected by the menstrual cycle. In comparison, the females‐BC group had poor reliability in their responses between the 2 days, though the specific effect of hormonal contraceptives is unknown. The difference between days in hypoxia as well as the PHC coefficient of variation during exercise were within the range of previously reported values (Koehle et al., 2005; Mann et al., 2022).

Hormone changes are variable across the menstrual cycle as well as between and within females, with one study showing an estrogen range of 50 ng/mL during the luteal phase in different females (MacNutt et al., 2012). Research on the effects of progesterone have shown increases in ventilation at rest (England & Farhi, 1976; Slatkovska et al., 2006) and during exercise (Schoene et al., 1981); however, the menstrual cycle has minimal effect on cardiorespiratory values (Itoh et al., 2007). The alteration in ventilation from progesterone does not affect performance of maximal exercise or shorter exercise durations (Janse De Jonge, 2003). The lack of difference in the exercise chemoresponse suggests that the physiological response associated with exercise has a stronger effect than that of hormone fluctuations. Though some previous work has shown an increased chemoresponse in the luteal phase (Dombovy et al., 1987; Schoene et al., 1981), it is possible that our results differ due to the type of test used. Previously used rebreathing tests involved continuous increases in CO_2_ until the test was terminated. Although providing a large stimulus (P_ET_CO_2_ upwards of 65 mmHg), these tests have shown to reduce the tissue‐arterial CO_2_ gradient in brain tissue which reduces the influence of cerebral blood flow on the central chemoresponse (Carr et al., 2023). Therefore, an increase in P_ET_CO_2_ greater than +7 mmHg, which brought our participants between 48 and 54 mmHg, during steady state may be required to see a difference in the ventilatory slopes in females. Sex hormones have also been shown to impact the hypoxic response in animals exposed to both oestrogens, progestins, and testosterone (Bonekat et al., 1987; Tatsumi et al., 1993, 1994).

### 4.3 Peripheral chemosensitivity

The females‐BC had a lower response compared to the male participants; however, this effect did not persist when scaled for body surface area. We emphasize the influence of allometric scaling because sex differences in the PHC were also eliminated when scaled for body surface area in previous work (Mann et al., 2022; Wright et al., 2023). The response between the two female groups was different after allometric scaling, which suggests that the hormonal contraceptives were reducing the ventilatory response to the transient CO_2_ stimulus. There was also a relationship between day 2 and 3 of the transient hypercapnia for male participants but not for either female group. It is unclear why the females‐BC group had a lower peripheral exercise chemoresponse. It is possible that the background hormone concentrations could have caused an increased ventilation prior to the stimulus, as has been shown at rest (Smith & Mines, 1982), and thus the hypercapnia would not raise ventilation to a similar degree. However, as the females‐BC group had a lower starting ventilation for all conditions, this is unlikely. The normally menstruating participants' ventilation was not different on day 3, during the luteal phase, whereas other studies have seen a greater ventilation during the luteal phase (Dombovy et al., 1987; MacNutt et al., 2012; Schoene et al., 1981). Our results may differ from previous work due to being earlier in the luteal phase; however, only one study (MacNutt et al., 2012) measured hormone concentrations for each female participant, and they demonstrated that the hormone peaks were not consistently timed between participants.

We hypothesized that exercise could be overriding the influence of hormones as several studies have shown no impact of hormones on physiological responses, such as ventilation, during exercise (Bonekat et al., 1987; Carmichael et al., 2021; MacNutt et al., 2012; Smekal et al., 2007). The females‐NBC in our study showed no difference between day 2 (i.e., follicular phase), and day 3 (i.e., luteal phase). Though the exercise effect could explain why females‐NBC showed no differences, it still does not explain why females‐BC had an overall lower response. Given the large variability in the response, regardless of group, we cannot exclude the possibility that this finding is spurious and occurred due to chance.

Females‐NBC also had a lower response overall compared to males in hyperoxia. These differences in the response of the normally menstruating group are likely due to a greater variability, with a significantly higher coefficient of variation compared to the females‐BC. These results suggest that the tonic input of the peripheral chemoreceptors could be impacted by the fluctuating hormones. The specific mechanism of the hormones on the peripheral chemoreceptors to cause this variation is unknown.

There were no differences in the isocapnic hypoxic ventilatory response between groups or days, though there were relationships between days 2 and 3 for males and females‐NBC. Our results are consistent with other work, in that there was no difference in the hypoxic response between days (Koehle et al., 2005) as well as no influence of menstrual cycle or oral contraceptives (Loeppky et al., 2001; MacNutt et al., 2012). A recent study examining the resting hypoxic ventilatory response at sea level also found no differences in the response between the early follicular, late follicular, or mid‐luteal time points (Citherlet et al., 2024). When males have been treated with progesterone to experimentally manipulate hormonal effects, the results are mixed, with both an increase and no change in the ventilatory response to hypoxia (Bonekat et al., 1987; Okita et al., 1987) but neither showing an overall effect on exercise performance. Thus, it is likely that the exercise responses would supersede any hormonal influence on the hypoxic response. Animal research has suggested that both male and female sex hormones impact the peripheral chemoreceptors by increasing the response to hypoxia and hypercapnia (Marques et al., 2015; Tatsumi et al., 1993, 1994, 1997). The similar influence of both male and female hormones over the response to hypoxia is consistent with our results showing no difference in the response between males and females.

### 4.4 Central chemosensitivity

The central chemoresponse to steady state iso‐oxic hypercapnia was unaffected by menstrual phase, nor was it different between males, females‐NBC, and females‐BC during exercise. These findings add to the mixed results of previous work, which observed both an increased response (Dombovy et al., 1987; Schoene et al., 1981) and no difference in response (MacNutt et al., 2012). The lack of difference between the responses of each group could be attributed to the exercise response, as previous research has shown that exercise causes an increase in the peripheral response to CO_2_ (Mann et al., 2022; Wright et al., 2023). Since the participants were kept iso‐oxic and not hyperoxic (as in the previous rebreathing studies), there would have been contribution by the peripheral chemoreceptors that would have impacted the overall response. However, the peripheral response to hypercapnia was not different within each group and would have led to a similar contribution between days. The male participants also showed a relationship between their day 2 and 3 responses, showing a greater reproducibility, where both female groups showed no relationship. Although others (Assadpour et al., 2020) have shown that high hormone phases increased the response to steady state 5% CO_2_, as indicated by enhanced chemoreflex, our findings suggest that this effect is diminished due to exercise as seen in previous work (MacNutt et al., 2012).

### 4.5 Implications

Given our relatively small sample size, our findings should not be interpreted on a population basis. Rather, our goal was to highlight that in the physiological context of the control of exercise hyperpnea, different days of the menstrual cycle do not appear to have a greater impact beyond that of typical biological variability. We highlight this because previously females have been excluded from a significant portion of research i part due to unknown influences of the menstrual cycle (Cowan et al., 2023), or were only tested during the early follicular phase when hormone levels are lower. As a result of decades of exclusion, there has been limited research on females compared to males. Our specific study showed no significant effect of the menstrual cycle on the day‐to‐day variability of the submaximal exercise chemoresponses. Our data supports the consideration of utilizing greater flexibility of testing protocols for future studies, as opposed to choosing only the follicular phase. These results also highlight that hormonal contraceptives could be impacting the physiological response to transient CO_2_ differently to naturally occurring hormones in a way that is not well understood currently through animal research.

### 4.6 Technical considerations and limitations

Our study has several technical considerations to be discussed. First, hypoxic end‐tidal forcing was done based on oxyhaemoglobin saturation instead of P_ET_O_2_. During exercise, and especially in hypoxia, the alveolar‐to‐arterial O_2_ gradient widens to a variable degree (Stickland et al., 2013); thus, P_ET_O_2_ becomes less reflective of arterial oxygen tensions. Since the peripheral chemoreceptors sense arterial oxygen tensions, it is possible the stimulus was not consistent between days. However, we kept SpO_2_ consistent between days. Low intensity exercise is unlikely to elicit pH or temperature changes (and the hypoxia was isocapnic), which ensured that the oxygen tension to oxyhaemoglobin saturation relationship (i.e., the oxygen dissociation curve) remains unchanged and stable. Secondly, ovulations kits were used to detect a spike in hormone concentration; however, this approach does not provide an absolute hormone concentration, and we did not directly measure hormone concentration because we were not interested in how absolute concentration of hormones impacted the exercise chemoresponses in a dose‐dependent relationship. Instead, we were interested in the changes between days within a subject. Similarly, the different types of contraceptives used in the females‐BC group would lead to differing levels of circulating hormones. However, as previously highlighted, our primary interest was between day comparisons within subjects. Thus, while each subject's hormone concentration likely differed, they would be similar between days in the females‐BC group. The females‐NBC group had a significantly lower peak heart rate compared to the other two groups. The females‐NBC group also had a nonsignificant lower VO_2_ during maximal exercise compared to the females‐BC group. It is possible that the females‐NBC group was either less aerobically trained than the females‐BC group or they may not have reached maximal intensities. However, this limitation is unlikely to impact our main outcomes (chemosensitivity during low intensity exercise), as the maximal exercise test only provided workloads for subsequent days. Furthermore, we have recently shown that hypercapnic ventilatory sensitivity does not depend on exercise intensity (Thompson et al., 2024; Wright et al., 2023). Lastly, we acknowledge that our relatively small sample size precludes generalization to the population level with regards to the effect of the menstrual phases influence on chemosensitivity during exercise.

## 5 CONCLUSION

We sought to address whether the sensitivity of the central and peripheral chemoreceptors during exercise was influenced by the menstrual cycle. We found no differences in the response to hypercapnia, hyperoxia, or hypoxia during exercise between the follicular and luteal phases in normally menstruating females. There were also no differences between two similarly spaced time points in females on hormonal contraceptives or males during submaximal aerobic exercise. We interpret this finding to mean that the normal biological variation in chemosensitivity during submaximal aerobic exercise is greater than the effects exerted by the fluctuations in hormones across the menstrual cycle or hormonal contraceptives. As such, studies investigating exercise hyperpnea that are not specifically interested in menstrual cycle effects can and should include normally menstruating female participants and those using hormonal contraceptives as the day‐to‐day variability is not significantly different from males.

## AUTHOR CONTRIBUTIONS

LMM, JSC, and PBD were involved in experimental design. LMM, MDW, BPT, JCC, JSC, and PBD were involved in data acquisition. LMM, MDW, and PBD were involved in data analysis. All authors interpreted, drafted, and approved the final version. All authors agree to be accountable for all aspects of the work in ensuring that questions related to the accuracy or integrity of any part of the work are appropriately investigated and resolved. All persons designated as authors qualify for authorship, and all those who qualify for authorship are listed.

## FUNDING INFORMATION

This work was supported by the Natural Sciences and Engineering Research Council of Canada (NSERC) (RGPIN‐2019‐04615) and an infrastructure grant from the Canada Foundation for Innovation (#38432).

## Data Availability

The data that support the findings on this study are available from the corresponding author upon reasonable request.

## References

[phy270169-bib-0001] Assadpour, E. , Ivry, I. , Wasef, S. , Adeyinka, B. , Murray, K. R. , & Edgell, H. (2020). Oral contraceptives and menstrual cycle influence autonomic reflex function. Physiological Reports, 8, 1–13. 10.14814/phy2.14550 PMC7507440 32889781

[phy270169-bib-0002] Blain, G. M. , Smith, C. A. , Henderson, K. S. , & Dempsey, J. A. (2009). Contribution of the carotid body chemoreceptors to eupneic ventilation in the intact, unanesthetized dog. Journal of Applied Physiology, 106, 1564–1573. 19246650 10.1152/japplphysiol.91590.2008 PMC2681333

[phy270169-bib-0003] Bonekat, H. W. , Dombovy, M. L. , & Staats, B. A. (1987). Progesterone‐induced changes in exercise performance and ventilatory response. Medicine and Science in Sports and Exercise, 19, 118–123. 2952862

[phy270169-bib-0004] Carmichael, M. A. , Thomson, R. L. , Moran, L. J. , & Wycherley, T. P. (2021). The impact of menstrual cycle phase on athletes' performance: A narrative review. International Journal of Environmental Research and Public Health, 18, 1–24. 10.3390/ijerph18041667 PMC7916245 33572406

[phy270169-bib-0005] Carr, J. M. J. R. , Day, T. A. , Ainslie, P. N. , & Hoiland, R. L. (2023). The jugular venous‐to‐arterial difference during rebreathing and end‐tidal forcing: Relationship with cerebral perfusion. The Journal of Physiology, 601, 4251–4262. 37635691 10.1113/JP284449

[phy270169-bib-0006] Citherlet, T. , Raberin, A. , Manferdelli, G. , Pialoux, V. , & Millet, G. P. (2024). Menstrual cycle does not impact the hypoxic ventilatory response and acute mountain sickness prediction. Scientific Reports, 14, 26087. 39477965 10.1038/s41598-024-76404-y PMC11525676

[phy270169-bib-0007] Cowan, S. M. , Kemp, J. L. , Ardern, C. L. , Thornton, J. S. , Rio, E. K. , Bruder, A. M. , Mosler, A. B. , Patterson, B. , Haberfield, M. , Roughead, E. A. , Hart, H. , To, L. , Neufeld, S. , Mazahir, N. , & Crossley, K. M. (2023). Sport and exercise medicine/physiotherapy publishing has a gender/sex equity problem: We need action now! British Journal of Sports Medicine, 57, 401–407. 36631242 10.1136/bjsports-2022-106055

[phy270169-bib-0008] de Oliveira, D. M. , Lopes, T. R. , Gomes, F. S. , Rashid, A. , & Silva, B. M. (2023). Ventilatory response to peripheral chemoreflex and muscle metaboreflex during static handgrip in healthy humans: Evidence of hyperadditive integration. Experimental Physiology, 108, 932–939. 37036125 10.1113/EP091094 PMC10988439

[phy270169-bib-0009] Dombovy, M. L. , Bonekat, H. W. , Williams, T. J. , & Staats, B. A. (1987). Exercise performance and ventilatory response in the menstrual cycle. Medicine and Science in Sports and Exercise, 19, 111–117. 3574043

[phy270169-bib-0010] Edgell, H. , & Stickland, M. K. (2014). Activation of the carotid chemoreflex secondary to muscle metaboreflex stimulation in men. American Journal of Physiology—Regulatory, Integrative and Comparative Physiology, 306, 693–700. 10.1152/ajpregu.00472.2013 24573180

[phy270169-bib-0011] England, S. J. , & Farhi, L. E. (1976). Fluctuations in alveolar CO2 and in base excess during the menstrual cycle1. Respiration Physiology, 26, 157–161. 935695 10.1016/0034-5687(76)90093-1

[phy270169-bib-0012] Farra, S. D. , Kessler, C. , Duffin, J. , Wells, G. D. , & Jacobs, I. (2016). Clamping end‐tidal carbon dioxide during graded exercise with control of inspired oxygen. Respiratory Physiology & Neurobiology, 231, 28–36. 27236039 10.1016/j.resp.2016.05.013

[phy270169-bib-0013] Hannhart, B. , Pickett, C. K. , & Moore, L. G. (1990). Effects of estrogen and progesterone on carotid body neural output responsiveness to hypoxia. Journal of Applied Physiology, 68, 1909–1916. 2113903 10.1152/jappl.1990.68.5.1909

[phy270169-bib-0014] Itoh, M. , Ueoka, H. , Aoki, T. , Hotta, N. , Kaneko, Y. , Takita, C. , & Fukuoka, Y. (2007). Exercise hyperpnea and hypercapnic ventilatory responses in women. Respiratory Medicine, 101, 446–452. 16934968 10.1016/j.rmed.2006.07.011

[phy270169-bib-0015] Janse De Jonge, X. A. K. (2003). Effects of the menstrual cycle on exercise performance. Sports Medicine, 33, 833–851. 12959622 10.2165/00007256-200333110-00004

[phy270169-bib-0016] Jensen, D. , Wolfe, L. A. , O'Donnell, D. E. , & Davies, G. A. L. (2005). Chemoreflex control of breathing during wakefulness in healthy men and women. Journal of Applied Physiology, 98, 822–828. 15557008 10.1152/japplphysiol.01208.2003

[phy270169-bib-0017] Koehle, M. S. , Foster, G. E. , McKenzie, D. C. , & Sheel, A. W. (2005). Repeated measurement of hypoxic ventilatory response as an intermittent hypoxic stimulus. Respiratory Physiology & Neurobiology, 145, 33–39. 15652786 10.1016/j.resp.2004.09.004

[phy270169-bib-0018] Loeppky, J. A. , Scotto, P. , Charlton, G. C. , Gates, L. , Icenogle, M. , & Roach, R. C. (2001). Ventilation is greater in women than men, but the increase during acute altitude hypoxia is the same. Respiration Physiology, 125, 225–237. 11282389 10.1016/s0034-5687(00)00221-8

[phy270169-bib-0019] MacNutt, M. J. , De Souza, M. J. , Tomczak, S. E. , Homer, J. L. , & Sheel, A. W. (2012). Resting and exercise ventilatory chemosensitivity across the menstrual cycle. Journal of Applied Physiology, 112, 737–747. 22174398 10.1152/japplphysiol.00727.2011

[phy270169-bib-0020] Mann, L. M. , Chan, J. S. , Angus, S. A. , Doherty, C. J. , Thompson, B. P. , Foster, G. E. , Hughson, R. L. , & Dominelli, P. B. (2022). Perihperal hypercapnic chemosensitivity in train and untrained females and males during exercise. Journal of Applied Physiology, 133, 1309–1317. 10.1152/japplphysiol.00460.2022 36302156

[phy270169-bib-0021] Marques, D. A. , de Carvalho, D. , Da Silva, G. S. F. , Szawka, R. E. , Anselmo‐Franci, J. A. , Bícego, K. C. , & Gargaglioni, L. H. (2015). Ventilatory, metabolic, and thermal responses to hypercapnia in female rats: Effects of estrous cycle, ovariectomy, and hormonal replacement. Journal of Applied Physiology, 119, 61–68. 25930026 10.1152/japplphysiol.00254.2015

[phy270169-bib-0023] Okita, S. , Kimura, H. , Kunitomo, F. , Tojima, H. , Yuguchi, Y. , Tatsumi, K. , Kuriyama, T. , Watanabe, S. , & Honda, Y. (1987). Effect of chlormadinone acetate, a synthetic progesterone, on hypoxic ventilatory response in men. The Japanese Journal of Physiology, 37, 137–147. 2441098 10.2170/jjphysiol.37.137

[phy270169-bib-0024] Schoene, R. B. , Robertson, H. T. , & Pierson, D. J. (1981). Respiratory drives and exercise in menstrual cycles of athletic and nonathletic women. Journal of Applied Physiology: Respiratory, Environmental and Exercise Physiology, 50, 1300–1305. 7263392 10.1152/jappl.1981.50.6.1300

[phy270169-bib-0025] Slatkovska, L. , Jensen, D. , Davies, G. A. L. , & Wolfe, L. A. (2006). Phasic menstrual cycle effects on the control of breathing in healthy women. Respiratory Physiology & Neurobiology, 154, 379–388. 16542884 10.1016/j.resp.2006.01.011

[phy270169-bib-0026] Smekal, G. , Von Duvillard, S. P. , Frigo, P. , Tegelhofer, T. , Pokan, R. , Hofmann, P. , Tschan, H. , Baron, R. , Wonisch, M. , Renezeder, K. , & Bachl, N. (2007). Menstrual cycle: No effect on exercise cardiorespiratory variables or blood lactate concentration. Medicine and Science in Sports and Exercise, 39, 1098–1106. 17596777 10.1249/mss.0b013e31805371e7

[phy270169-bib-0027] Smith, C. A. , & Mines, A. H. (1982). Ventilatory response of humans to chronic contraceptive pill administration. Respiration, 43, 179–185. 7111865 10.1159/000194483

[phy270169-bib-0028] Steinback, C. D. , & Poulin, M. J. (2007). Ventilatory responses to isocapnic and poikilocapnic hypoxia in humans. Respiratory Physiology & Neurobiology, 155, 104–113. 16815106 10.1016/j.resp.2006.05.006

[phy270169-bib-0029] Stickland, M. K. , Lindinger, M. I. , Olfert, I. M. , Heigenhauser, G. J. F. , & Hopkins, S. R. (2013). Pulmonary gas exchange and acid‐base balance during exercise. Comprehensive Physiology, 3, 693–739. 23720327 10.1002/cphy.c110048 PMC8315793

[phy270169-bib-0030] Tatsumi, K. , Hannhart, B. , Pickett, C. K. , Weil, J. V. , & Moore, L. G. (1994). Effects of testosterone on hypoxic ventilatory and carotid body neural responsiveness. American Journal of Respiratory and Critical Care Medicine, 149, 1248–1253. 8173766 10.1164/ajrccm.149.5.8173766

[phy270169-bib-0031] Tatsumi, K. , Mikami, M. , Kuriyama, T. , & Fukuda, Y. (1993). Effects of a synthetic progestin on ventilatory response to hypoxia in awake male rats. Nihon Kyōbu Shikkan Gakkai zasshi, 31, 563–568. 7687309

[phy270169-bib-0032] Tatsumi, K. , Pickett, C. K. , Jacoby, C. R. , Weil, J. V. , & Moore, L. G. (1997). Role of endogenous female hormones in hypoxic chemosensitivity. Journal of Applied Physiology, 83, 1706–1710. 9375342 10.1152/jappl.1997.83.5.1706

[phy270169-bib-0033] Taylor, M. Y. , Osborne, J. O. , Topranin, V. D. M. , Engseth, T. P. , Solli, G. S. , Valsdottir, D. , Andersson, E. , Øistuen, G. F. , Flatby, I. , Welde, B. , Morseth, B. , Haugen, T. , Sandbakk, Ø. , & Noordhof, D. A. (2024). Menstrual cycle phase has no influence on performance‐determining variables in endurance‐trained athletes: The FENDURA Project. Medicine and Science in Sports and Exercise, 56, 1595–1605. 38600646 10.1249/MSS.0000000000003447

[phy270169-bib-0034] Thompson, A. J. , Wright, M. D. , Mann, L. M. , Pulford‐Thorpe, A. E. , & Dominelli, P. B. (2024). Ventilatory response of peripheral chemoreceptors to hypercapnia during exercise above the respiratory compensation point. Journal of Applied Physiology, 137, 125–135. 38813610 10.1152/japplphysiol.00002.2024

[phy270169-bib-0035] Tremblay, M. S. , Warburton, D. E. R. , Janssen, I. , Paterson, D. H. , Latimer, A. E. , Rhodes, R. E. , Kho, M. E. , Hicks, A. , LeBlanc, A. G. , Zehr, L. , Murumets, K. , & Duggan, M. (2011). New Canadian physical activity guidelines. Applied Physiology, Nutrition, and Metabolism, 36, 36–46. 10.1139/H11-009 21326376

[phy270169-bib-0036] Tymko, M. M. , Ainslie, P. N. , Macleod, D. B. , Willie, C. K. , & Foster, G. E. (2015). End tidal‐to‐arterial CO2 and O2 gas gradients at low‐ and high‐altitude during dynamic end‐tidal forcing. American Journal of Physiology—Regulatory, Integrative and Comparative Physiology, 308, R895–R906. 25810386 10.1152/ajpregu.00425.2014

[phy270169-bib-0037] Wright, M. D. , Mann, L. M. , Doherty, C. J. , Thompson, B. P. , Angus, S. A. , Chang, J.‐C. , & Dominelli, P. B. (2023). Peripheral hypercapnic chemosensitivity at rest and progressive exercise intensities in males and females. Journal of Applied Physiology, 136, 274–282. 38126093 10.1152/japplphysiol.00578.2023

